# 1-{3-(4-Chloro­phen­yl)-5-[4-(propan-2-yl)phen­yl]-4,5-di­hydro-1*H*-pyrazol-1-yl}ethanone

**DOI:** 10.1107/S1600536814013348

**Published:** 2014-06-14

**Authors:** B. Narayana, Vinutha V. Salian, Balladka K. Sarojini, Jerry P. Jasinski

**Affiliations:** a Department of Studies in Chemistry, Mangalore University, Mangalagangotri 574 199, India; b Department of Studies in Chemistry, Industrial Chemistry Section, Mangalore University, Mangalagangotri 574 199, India; c Department of Chemistry, Keene State College, 229 Main Street, Keene, NH 03435-2001, USA

## Abstract

In the title compound, C_20_H_21_ClN_2_O, the dihedral angles between the pyrazole ring (r.m.s. deviation = 0.049 Å) and the benzene and chloro­benzene rings are 84.65 (10) and 3.35 (10)°, respectively. In the crystal, inversion dimers linked by pairs of weak C—H⋯O inter­actions generate *R*_2_^2^(16) loops. Weak π–π stacking inter­actions [centroid–centroid distance = 3.8490 (11) Å] are also observed.

## Related literature   

For background to pyrazolines, see: Manna *et al.* (2005 ▶); Samshuddin *et al.* (2012 ▶). For a related structure, see: Jasinski *et al.* (2010 ▶).
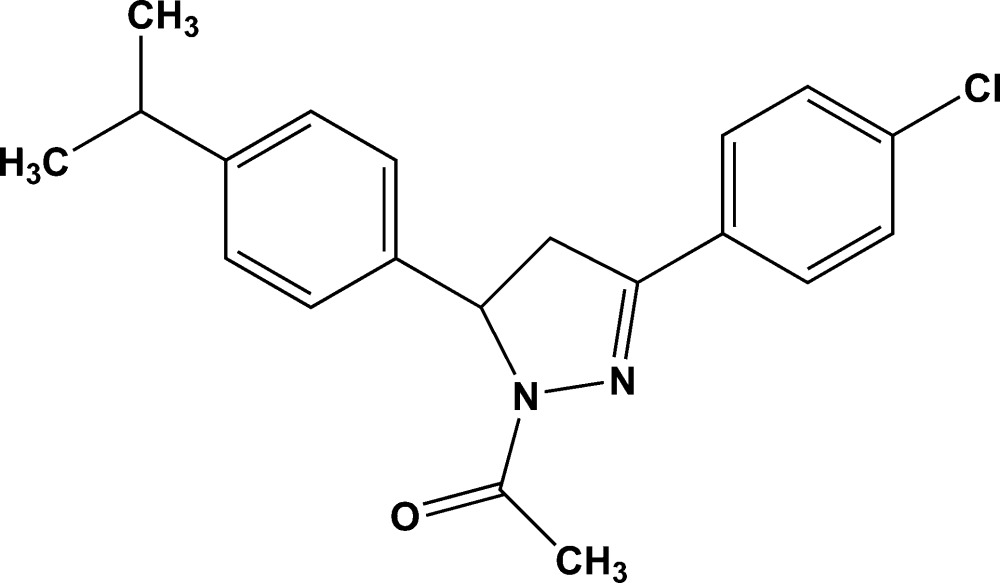


## Experimental   

### 

#### Crystal data  

C_20_H_21_ClN_2_O
*M* *_r_* = 340.84
Triclinic, 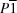

*a* = 6.4836 (6) Å
*b* = 9.6524 (9) Å
*c* = 14.439 (1) Å
α = 81.178 (7)°
β = 89.720 (7)°
γ = 77.488 (8)°
*V* = 871.35 (13) Å^3^
*Z* = 2
Cu *K*α radiation
μ = 2.00 mm^−1^
*T* = 173 K
0.44 × 0.22 × 0.12 mm

#### Data collection  

Agilent Eos Gemini diffractometer
Absorption correction: multi-scan (*CrysAlis PRO* and *CrysAlis RED*; Agilent, 2012 ▶) *T*_min_ = 0.552, *T*_max_ = 1.000
5081 measured reflections
3287 independent reflections
2770 reflections with *I* > 2σ(*I*)
*R*_int_ = 0.030

#### Refinement  

*R*[*F* ^2^ > 2σ(*F* ^2^)] = 0.049
*wR*(*F* ^2^) = 0.142
*S* = 1.03
3287 reflections
220 parameters
H-atom parameters constrained
Δρ_max_ = 0.39 e Å^−3^
Δρ_min_ = −0.30 e Å^−3^

### 

Data collection: *CrysAlis PRO* (Agilent, 2012 ▶); cell refinement: *CrysAlis PRO*; data reduction: *CrysAlis RED* (Agilent, 2012 ▶); program(s) used to solve structure: *SUPERFLIP* (Palatinus *et al.*, 2012 ▶); program(s) used to refine structure: *SHELXL2012* (Sheldrick, 2008 ▶); molecular graphics: *OLEX2* (Dolomanov *et al.*, 2009 ▶); software used to prepare material for publication: *OLEX2*.

## Supplementary Material

Crystal structure: contains datablock(s) I. DOI: 10.1107/S1600536814013348/hb7234sup1.cif

Structure factors: contains datablock(s) I. DOI: 10.1107/S1600536814013348/hb7234Isup2.hkl

Supporting information file. DOI: 10.1107/S1600536814013348/hb7234Isup3.cml

CCDC reference: 1007161

Additional supporting information:  crystallographic information; 3D view; checkCIF report

## Figures and Tables

**Table 1:** Hydrogen-bond geometry (Å, °)

*D*—H⋯*A*	*D*—H	H⋯*A*	*D*⋯*A*	*D*—H⋯*A*
C15—H15⋯O1^i^	0.95	2.44	3.364 (2)	165

Symmetry code: (i) 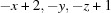.

**Fig. 1.**
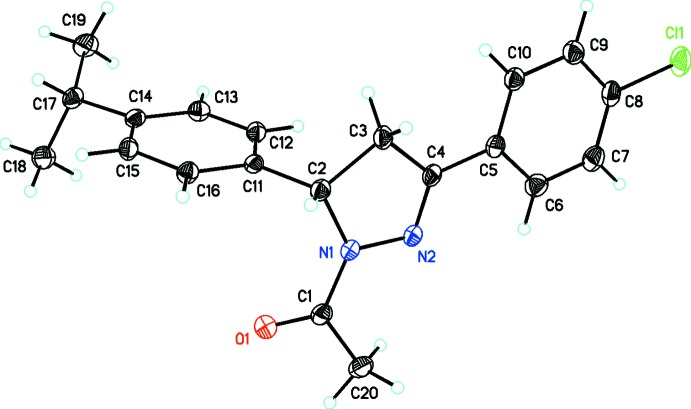
ORTEP drawing of (I), C20H21ClN2O, showing 30% probability displacement ellipsoids.

**Fig. 2.**
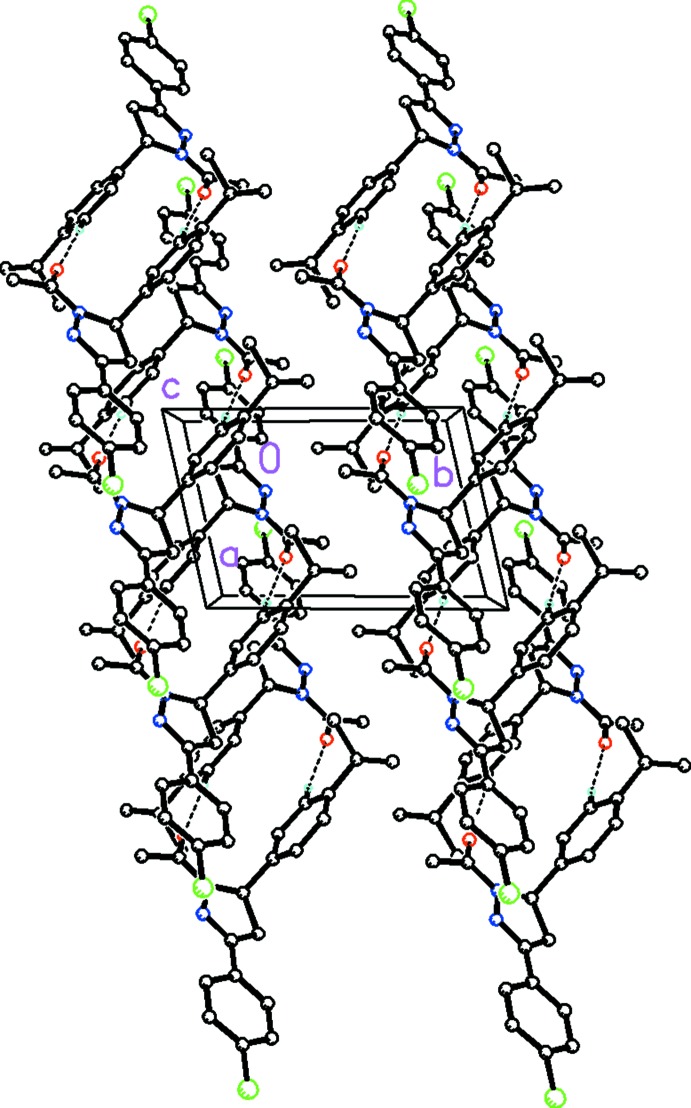
Molecular packing for (I) viewed along the c axis. Dashed lines indicate weak C—H···O interactions between the phenyl ring and the ethanone group forming dimers in an R22(16) ring motif stacked along the ab plane. H atoms not involved with these weak interactions have been removed for clarity.

**Fig. 3.**
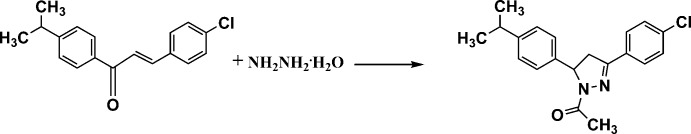
Synthesis of (I), C20H21ClN2O.

## supplementary crystallographic information

### S1. Comment

Pyrazoline derivatives possess many biological activities such as anticancer (Manna *et al.*, 2005) and antioxidant properties (Samshuddin *et al.*, 2012). As part of our ongoing studies in this area (Jasinski *et al.*, 2010), we now describe the structure of the title compound, C_20_H_21_ClN_2_O.

The dihedral angle between the mean planes of the phenyl rings is 81.3 (0)° while the pyrazole ring is separated from each of the phenyl rings by 3.3 (5)° (C5–C10) and 84.6 (5)° (C11–C16), respectively (Fig. 1). In the crystal, a weak C—H···O intermolecular interaction between the phenyl ring and the ethanone group is observed forming dimers in an R_2_^2^(16) ring-set motif stacked along the *ab* plane (Fig. 2). In addition, weak π–π intermolecular stacking interactions (Cg1–Cg3 = 3.8490 (11)Å, x, y, z, Cg1: N1/N2/C2/C3/C4; Cg3: C11–C16) are present.

### S2. Experimental

To a mixture of (2E)-1-(4-chlorophenyl)-3-[4-(propan-2-yl) phenyl] prop-2-en-1-one (2.85g, 0.01 mol) and hydrazine hydrate (0.5mL, 0.01 mol) in 25 mL acetic acid was refluxed for 9h (Fig. 3). The reaction mixture was cooled and poured into ice-cold water. The precipitate formed was collected by filtration and purified by recrystallization from ethanol. Colourless, irregular, crystals were grown from ethanol solution by the slow evaporation method (m.p.: 389–391 K).

### S3. Refinement

All of the H atoms were placed in their calculated positions and then refined using the riding model with Atom—H lengths of 0.95 - 1.00Å (CH), 0.99Å (CH_2_) or 0.98Å (CH_3_). Isotropic displacement parameters for these atoms were set to 1.2 (CH, CH_2_) or 1.5 (CH_3_) times *U*_eq_ of the parent atom. Idealised Me refined as a rotating group.

### Crystal data

**Table:**

C_20_H_21_ClN_2_O	*Z* = 2
*M**_r_* = 340.84	*F*(000) = 360
Triclinic, *P*1	*D*_x_ = 1.299 Mg m^−^^3^
*a* = 6.4836 (6) Å	Cu *K*α radiation, λ = 1.54184 Å
*b* = 9.6524 (9) Å	Cell parameters from 2061 reflections
*c* = 14.439 (1) Å	θ = 4.7–71.3°
α = 81.178 (7)°	µ = 2.00 mm^−^^1^
β = 89.720 (7)°	*T* = 173 K
γ = 77.488 (8)°	Irregular, colourless
*V* = 871.35 (13) Å^3^	0.44 × 0.22 × 0.12 mm

### Data collection

**Table:**

Agilent Eos Gemini diffractometer	3287 independent reflections
Radiation source: Enhance (Cu) X-ray Source	2770 reflections with *I* > 2σ(*I*)
Graphite monochromator	*R*_int_ = 0.030
Detector resolution: 16.0416 pixels mm^-1^	θ_max_ = 71.3°, θ_min_ = 4.8°
ω scans	*h* = −7→7
Absorption correction: multi-scan (*CrysAlis PRO* and *CrysAlis RED*; Agilent, 2012)	*k* = −11→11
*T*_min_ = 0.552, *T*_max_ = 1.000	*l* = −17→13
5081 measured reflections	

### Refinement

**Table:**

Refinement on *F*^2^	Primary atom site location: structure-invariant direct methods
Least-squares matrix: full	Hydrogen site location: inferred from neighbouring sites
*R*[*F*^2^ > 2σ(*F*^2^)] = 0.049	H-atom parameters constrained
*wR*(*F*^2^) = 0.142	*w* = 1/[σ^2^(*F*_o_^2^) + (0.0831*P*)^2^ + 0.1082*P*] where *P* = (*F*_o_^2^ + 2*F*_c_^2^)/3
*S* = 1.03	(Δ/σ)_max_ < 0.001
3287 reflections	Δρ_max_ = 0.39 e Å^−^^3^
220 parameters	Δρ_min_ = −0.30 e Å^−^^3^
0 restraints	

### Special details

**Table:**

Geometry. All esds (except the esd in the dihedral angle between two l.s. planes) are estimated using the full covariance matrix. The cell esds are taken into account individually in the estimation of esds in distances, angles and torsion angles; correlations between esds in cell parameters are only used when they are defined by crystal symmetry. An approximate (isotropic) treatment of cell esds is used for estimating esds involving l.s. planes.

### Fractional atomic coordinates and isotropic or equivalent isotropic displacement parameters (Å^2^)

**Table:**

	*x*	*y*	*z*	*U*_iso_*/*U*_eq_	
Cl1	−0.38093 (9)	0.20917 (6)	−0.11269 (4)	0.0519 (2)	
O1	0.7663 (2)	0.28686 (15)	0.34910 (10)	0.0410 (4)	
N1	0.5281 (2)	0.23124 (16)	0.25819 (10)	0.0274 (3)	
N2	0.4029 (2)	0.26002 (16)	0.17744 (10)	0.0281 (3)	
C1	0.6681 (3)	0.31158 (19)	0.27431 (14)	0.0312 (4)	
C2	0.4721 (3)	0.11838 (19)	0.33008 (12)	0.0270 (4)	
H2	0.4340	0.1587	0.3892	0.032*	
C3	0.2714 (3)	0.0933 (2)	0.28392 (12)	0.0296 (4)	
H3A	0.2847	−0.0094	0.2789	0.036*	
H3B	0.1441	0.1262	0.3196	0.036*	
C4	0.2629 (3)	0.18369 (18)	0.18828 (12)	0.0270 (4)	
C5	0.1068 (3)	0.18983 (19)	0.11335 (12)	0.0286 (4)	
C6	0.0992 (3)	0.2857 (2)	0.02914 (13)	0.0367 (4)	
H6	0.1983	0.3456	0.0193	0.044*	
C7	−0.0529 (3)	0.2928 (2)	−0.03959 (14)	0.0398 (5)	
H7	−0.0602	0.3586	−0.0962	0.048*	
C8	−0.1938 (3)	0.2031 (2)	−0.02500 (14)	0.0357 (4)	
C9	−0.1890 (3)	0.1079 (2)	0.05667 (14)	0.0353 (4)	
H9	−0.2875	0.0474	0.0657	0.042*	
C10	−0.0374 (3)	0.1018 (2)	0.12568 (13)	0.0310 (4)	
H10	−0.0325	0.0362	0.1823	0.037*	
C11	0.6507 (3)	−0.01311 (17)	0.35070 (11)	0.0238 (4)	
C12	0.7114 (3)	−0.10535 (19)	0.28520 (12)	0.0281 (4)	
H12	0.6388	−0.0860	0.2261	0.034*	
C13	0.8759 (3)	−0.22467 (19)	0.30517 (13)	0.0291 (4)	
H13	0.9145	−0.2856	0.2592	0.035*	
C14	0.9860 (3)	−0.25741 (18)	0.39120 (12)	0.0264 (4)	
C15	0.9230 (3)	−0.16548 (19)	0.45676 (12)	0.0294 (4)	
H15	0.9934	−0.1859	0.5164	0.035*	
C16	0.7603 (3)	−0.04536 (19)	0.43676 (12)	0.0281 (4)	
H16	0.7227	0.0162	0.4824	0.034*	
C17	1.1712 (3)	−0.38433 (19)	0.41496 (13)	0.0310 (4)	
H17	1.1640	−0.4224	0.4831	0.037*	
C18	1.3796 (3)	−0.3346 (2)	0.40136 (17)	0.0429 (5)	
H18A	1.3855	−0.2630	0.4420	0.064*	
H18B	1.3885	−0.2919	0.3358	0.064*	
H18C	1.4982	−0.4172	0.4176	0.064*	
C19	1.1692 (3)	−0.5078 (2)	0.36083 (17)	0.0432 (5)	
H19A	1.1930	−0.4775	0.2944	0.065*	
H19B	1.0319	−0.5347	0.3672	0.065*	
H19C	1.2813	−0.5905	0.3861	0.065*	
C20	0.6915 (3)	0.4311 (2)	0.19747 (15)	0.0405 (5)	
H20A	0.8279	0.4566	0.2055	0.061*	
H20B	0.5770	0.5151	0.2004	0.061*	
H20C	0.6847	0.3993	0.1365	0.061*	

### Atomic displacement parameters (Å^2^)

**Table:**

	*U*^11^	*U*^22^	*U*^33^	*U*^12^	*U*^13^	*U*^23^
Cl1	0.0532 (4)	0.0469 (3)	0.0508 (3)	0.0024 (3)	−0.0251 (3)	−0.0103 (2)
O1	0.0397 (8)	0.0346 (8)	0.0482 (8)	−0.0092 (6)	−0.0126 (7)	−0.0028 (6)
N1	0.0275 (8)	0.0219 (7)	0.0303 (8)	−0.0028 (6)	−0.0031 (6)	−0.0005 (6)
N2	0.0270 (8)	0.0227 (7)	0.0318 (8)	−0.0004 (6)	−0.0019 (6)	−0.0023 (6)
C1	0.0268 (9)	0.0221 (8)	0.0428 (11)	−0.0001 (7)	0.0004 (8)	−0.0062 (7)
C2	0.0247 (9)	0.0246 (8)	0.0295 (9)	−0.0022 (7)	0.0013 (7)	−0.0014 (7)
C3	0.0219 (8)	0.0311 (9)	0.0320 (9)	−0.0020 (7)	−0.0002 (7)	0.0018 (7)
C4	0.0231 (8)	0.0231 (8)	0.0311 (9)	0.0024 (7)	0.0025 (7)	−0.0035 (7)
C5	0.0262 (9)	0.0262 (9)	0.0301 (9)	0.0014 (7)	0.0005 (7)	−0.0050 (7)
C6	0.0410 (11)	0.0344 (10)	0.0332 (10)	−0.0085 (9)	−0.0002 (8)	−0.0009 (8)
C7	0.0470 (12)	0.0364 (10)	0.0313 (10)	−0.0023 (9)	−0.0053 (9)	−0.0002 (8)
C8	0.0330 (10)	0.0325 (10)	0.0377 (10)	0.0050 (8)	−0.0082 (8)	−0.0108 (8)
C9	0.0310 (10)	0.0327 (10)	0.0410 (10)	−0.0025 (8)	−0.0024 (8)	−0.0082 (8)
C10	0.0274 (9)	0.0281 (9)	0.0343 (9)	−0.0005 (7)	0.0002 (7)	−0.0030 (7)
C11	0.0220 (8)	0.0203 (8)	0.0282 (8)	−0.0037 (7)	0.0006 (7)	−0.0022 (6)
C12	0.0288 (9)	0.0278 (9)	0.0268 (8)	−0.0050 (7)	−0.0029 (7)	−0.0033 (7)
C13	0.0313 (9)	0.0254 (9)	0.0313 (9)	−0.0039 (7)	0.0037 (7)	−0.0094 (7)
C14	0.0237 (8)	0.0206 (8)	0.0340 (9)	−0.0041 (7)	0.0025 (7)	−0.0026 (7)
C15	0.0305 (9)	0.0277 (9)	0.0277 (9)	−0.0026 (7)	−0.0054 (7)	−0.0028 (7)
C16	0.0305 (9)	0.0245 (8)	0.0283 (9)	−0.0027 (7)	0.0004 (7)	−0.0061 (7)
C17	0.0279 (9)	0.0246 (9)	0.0371 (10)	−0.0006 (7)	0.0026 (8)	−0.0019 (7)
C18	0.0279 (10)	0.0339 (10)	0.0636 (14)	−0.0024 (8)	−0.0021 (9)	−0.0036 (10)
C19	0.0379 (11)	0.0246 (9)	0.0647 (14)	0.0023 (8)	0.0018 (10)	−0.0126 (9)
C20	0.0400 (11)	0.0263 (9)	0.0536 (13)	−0.0074 (8)	0.0014 (9)	−0.0006 (9)

### Geometric parameters (Å, º)

**Table:**

Cl1—C8	1.744 (2)	C11—C12	1.395 (2)
O1—C1	1.221 (2)	C11—C16	1.393 (2)
N1—N2	1.381 (2)	C12—H12	0.9500
N1—C1	1.357 (2)	C12—C13	1.384 (2)
N1—C2	1.489 (2)	C13—H13	0.9500
N2—C4	1.282 (2)	C13—C14	1.394 (3)
C1—C20	1.505 (3)	C14—C15	1.395 (2)
C2—H2	1.0000	C14—C17	1.516 (2)
C2—C3	1.546 (2)	C15—H15	0.9500
C2—C11	1.514 (2)	C15—C16	1.382 (2)
C3—H3A	0.9900	C16—H16	0.9500
C3—H3B	0.9900	C17—H17	1.0000
C3—C4	1.510 (2)	C17—C18	1.531 (3)
C4—C5	1.471 (3)	C17—C19	1.524 (3)
C5—C6	1.405 (3)	C18—H18A	0.9800
C5—C10	1.387 (3)	C18—H18B	0.9800
C6—H6	0.9500	C18—H18C	0.9800
C6—C7	1.386 (3)	C19—H19A	0.9800
C7—H7	0.9500	C19—H19B	0.9800
C7—C8	1.383 (3)	C19—H19C	0.9800
C8—C9	1.376 (3)	C20—H20A	0.9800
C9—H9	0.9500	C20—H20B	0.9800
C9—C10	1.389 (3)	C20—H20C	0.9800
C10—H10	0.9500		
			
N2—N1—C2	113.23 (13)	C16—C11—C2	120.39 (15)
C1—N1—N2	122.58 (15)	C16—C11—C12	117.85 (15)
C1—N1—C2	123.64 (15)	C11—C12—H12	119.5
C4—N2—N1	108.69 (14)	C13—C12—C11	120.92 (16)
O1—C1—N1	120.05 (17)	C13—C12—H12	119.5
O1—C1—C20	122.85 (18)	C12—C13—H13	119.3
N1—C1—C20	117.09 (17)	C12—C13—C14	121.44 (16)
N1—C2—H2	109.5	C14—C13—H13	119.3
N1—C2—C3	100.98 (14)	C13—C14—C15	117.33 (16)
N1—C2—C11	112.19 (14)	C13—C14—C17	123.21 (16)
C3—C2—H2	109.5	C15—C14—C17	119.44 (16)
C11—C2—H2	109.5	C14—C15—H15	119.3
C11—C2—C3	114.97 (15)	C16—C15—C14	121.43 (16)
C2—C3—H3A	111.2	C16—C15—H15	119.3
C2—C3—H3B	111.2	C11—C16—H16	119.5
H3A—C3—H3B	109.2	C15—C16—C11	121.02 (16)
C4—C3—C2	102.59 (15)	C15—C16—H16	119.5
C4—C3—H3A	111.2	C14—C17—H17	107.3
C4—C3—H3B	111.2	C14—C17—C18	110.08 (15)
N2—C4—C3	114.03 (16)	C14—C17—C19	114.25 (16)
N2—C4—C5	121.42 (16)	C18—C17—H17	107.3
C5—C4—C3	124.53 (16)	C19—C17—H17	107.3
C6—C5—C4	120.82 (17)	C19—C17—C18	110.38 (16)
C10—C5—C4	120.14 (16)	C17—C18—H18A	109.5
C10—C5—C6	119.03 (17)	C17—C18—H18B	109.5
C5—C6—H6	120.0	C17—C18—H18C	109.5
C7—C6—C5	119.98 (19)	H18A—C18—H18B	109.5
C7—C6—H6	120.0	H18A—C18—H18C	109.5
C6—C7—H7	120.3	H18B—C18—H18C	109.5
C8—C7—C6	119.40 (19)	C17—C19—H19A	109.5
C8—C7—H7	120.3	C17—C19—H19B	109.5
C7—C8—Cl1	119.34 (16)	C17—C19—H19C	109.5
C9—C8—Cl1	118.93 (17)	H19A—C19—H19B	109.5
C9—C8—C7	121.73 (18)	H19A—C19—H19C	109.5
C8—C9—H9	120.6	H19B—C19—H19C	109.5
C8—C9—C10	118.71 (19)	C1—C20—H20A	109.5
C10—C9—H9	120.6	C1—C20—H20B	109.5
C5—C10—C9	121.13 (18)	C1—C20—H20C	109.5
C5—C10—H10	119.4	H20A—C20—H20B	109.5
C9—C10—H10	119.4	H20A—C20—H20C	109.5
C12—C11—C2	121.76 (15)	H20B—C20—H20C	109.5
			
Cl1—C8—C9—C10	178.96 (14)	C3—C4—C5—C6	174.73 (17)
N1—N2—C4—C3	2.3 (2)	C3—C4—C5—C10	−4.4 (3)
N1—N2—C4—C5	−179.36 (14)	C4—C5—C6—C7	−178.18 (17)
N1—C2—C3—C4	6.52 (17)	C4—C5—C10—C9	178.66 (16)
N1—C2—C11—C12	−69.6 (2)	C5—C6—C7—C8	−1.1 (3)
N1—C2—C11—C16	110.32 (18)	C6—C5—C10—C9	−0.5 (3)
N2—N1—C1—O1	175.76 (16)	C6—C7—C8—Cl1	−178.48 (15)
N2—N1—C1—C20	−3.2 (3)	C6—C7—C8—C9	0.8 (3)
N2—N1—C2—C3	−6.06 (18)	C7—C8—C9—C10	−0.3 (3)
N2—N1—C2—C11	116.86 (15)	C8—C9—C10—C5	0.2 (3)
N2—C4—C5—C6	−3.5 (3)	C10—C5—C6—C7	0.9 (3)
N2—C4—C5—C10	177.42 (16)	C11—C2—C3—C4	−114.46 (16)
C1—N1—N2—C4	−169.07 (16)	C11—C12—C13—C14	0.3 (3)
C1—N1—C2—C3	165.62 (16)	C12—C11—C16—C15	−0.5 (3)
C1—N1—C2—C11	−71.5 (2)	C12—C13—C14—C15	0.4 (3)
C2—N1—N2—C4	2.71 (19)	C12—C13—C14—C17	−178.11 (16)
C2—N1—C1—O1	4.8 (3)	C13—C14—C15—C16	−1.1 (3)
C2—N1—C1—C20	−174.12 (16)	C13—C14—C17—C18	98.9 (2)
C2—C3—C4—N2	−5.9 (2)	C13—C14—C17—C19	−26.0 (2)
C2—C3—C4—C5	175.76 (15)	C14—C15—C16—C11	1.1 (3)
C2—C11—C12—C13	179.65 (16)	C15—C14—C17—C18	−79.6 (2)
C2—C11—C16—C15	179.65 (16)	C15—C14—C17—C19	155.55 (18)
C3—C2—C11—C12	45.1 (2)	C16—C11—C12—C13	−0.2 (3)
C3—C2—C11—C16	−135.05 (17)	C17—C14—C15—C16	177.47 (16)

### Hydrogen-bond geometry (Å, º)

**Table:**

*D*—H···*A*	*D*—H	H···*A*	*D*···*A*	*D*—H···*A*
C15—H15···O1^i^	0.95	2.44	3.364 (2)	165

Symmetry code: (i) −*x*+2, −*y*, −*z*+1.
